# Intravenous immunoglobulin treatment of congenital parvovirus B19 induced anemia - a case report

**DOI:** 10.1186/s40748-023-00164-2

**Published:** 2023-08-07

**Authors:** Stephanie T. Aronson, Mahmut Y. Celiker, Ludovico Guarini, Rabia Agha

**Affiliations:** 1 Department of Pediatrics, Maimonides Children’s Hospital, 4802 10th Avenue, 11219 Brooklyn, NY USA; 2 Division of Pediatric Hematology/Oncology Department of Pediatrics, Maimonides Children’s Hospital, 4802 10th Avenue, 11219 Brooklyn, NY USA; 3 Division of Pediatric Infectious Diseases, Department of Pediatrics, Maimonides Children’s Hospital, 4802 10th Avenue, 11219 Brooklyn, NY USA

**Keywords:** IVIG, Perinatal infection, Parvovirus B19, Congenital parvovirus infection, Congenital anemia, Fetal anemia, Hydrops fetalis, Neonatal anemia

## Abstract

**Background:**

Parvovirus is a common childhood infection that could be very dangerous to the fetus, if pregnant women become infected. The spectrum of effects range from pure red blood cell aplasia with hydrops fetalis to meningoencephalitis, with many symptoms in between. Severe anemia in the setting of pure red blood cell aplasia is one of the more common effects that neonatal experience (if infected intrapartum), with the current gold standard treatment being intrauterine or postnatal packed red blood cell (PRBC) transfusions, yet intravenous immunoglobulin (IVIG) may be a superior treatment option.

**Case presentation:**

A preterm infant was born at 26th week of gestational age via emergency Cesarean section due to hydrops fetalis, with parvovirus B19 exposure one month prior. The infant tested positive for IgM antibodies against parvovirus B19. Among many other serious complications of both hydrops fetalis and premature delivery, the infant had severe unremitting anemia, and received many PRBC transfusion over the course of his 71-day-long neonatal intensive care unit stay. During a follow up appointments as outpatient, his blood tests showed persistent high copies of parvovirus B19. He was then supported with PRBC transfusions and treated with IVIG. After three doses of IVIG, the infant’s parvovirus B19 viral copy numbers have dramatically reduced and the infant did not require any more PRBC transfusions.

**Conclusions:**

IVIG infusion effectively treated the parvovirus B19 infection and restored erythropoiesis making the child transfusion independent. Furthermore, since IVIG is safe and readily crosses the placenta, further studies are needed to determine if IVIG should be considered as an alternative prenatal treatment for congenital parvovirus B19 infection.

## Background

Parvovirus B19 is a common cause of childhood infections mostly presenting in immunocompetent children as an intense red facial rash known as erythema infectiosum [1]. However, in the event that a pregnant woman becomes infected with parvovirus B19, the effects on the fetus have the possibility of causing serious disease [2–5].

Although parvovirus has minimal to no adverse effects on a healthy pregnant mother, it is transmitted via the placenta to the fetus. The transmission rate to fetus is about 33%, and the risk for fetal death can be as high as 10% in most studies except a Danish population study where they found no association between maternal parvovirus infection and disease in the infants [2, 4, 6–8]. The virus suppresses erythroid precursors in the early stages of hematopoiesis, which may lead to pure red blood cell aplasia and severe anemia [9]. If the infant of an affected mother survives, he/she may develop thrombocytopenia, myocarditis, hepatitis, vasculitis, meningoencephalitis, or sepsis-like syndrome as well [2].

Here we report a case of severe congenital anemia secondary to gestational parvovirus B19 infection, review of current knowledge on the management of parvovirus induced congenital anemia and use of intravenous immunoglobulins (IVIG) to treat the infection and resultant anemia.

## Case presentation

The child was born at an outside institution at 26th week of gestational age via emergency cesarean section secondary to hydrops fetalis to a 24-year-old mother. This was her second child and mother reported her older child having symptoms consistent with parvovirus infection 1 month prior to delivery. Mother’s evaluation at time of delivery showed presence of parvovirus B19 IgG antibodies but IgM antibodies were not detected.

Hydrops fetalis was thought to be related to congenital parvovirus infection. Percutaneous umbilical cord blood sampling showed hemoglobin of 3.1 g/dL and platelet count of 12,000/µL. An attempt to give in-utero-transfusion failed and emergency delivery was carried out. Baby was born with Apgar scores of 2, 6, and 6 at 1, 5, and 10 min of life, respectively. He was then transferred to neonatal intensive care unit (NICU) started on transfusion support with packed red blood cells (PRBC).

In the NICU, child was found to have positive IgM antibodies for parvovirus B19 but IgG was negative. A polymerase chain reaction (PCR) assay done on blood for parvovirus B19 showed 7.7 10^9^ IU/mL copy number. His neonatal course was complicated by grade 2 intraventricular hemorrhage and abdominal wall defect. He required extensive transfusion support and antibiotics for suspected sepsis in addition to respiratory and nutritional support. He was transferred to the neonatal intensive care unit of our institution at the chronologic age of 56 days. He continued to require PRBC transfusion support while in our NICU and was finally discharged home at 71 days of age.

At 121 days of life, the child presented to the emergency room with severe anemia and cardiac failure. He received PRBC transfusion in addition to cardiac and respiratory support and fully recovered from the event. He was sent home 5 days after admission with close follow-up by our Pediatric Hematology/Oncology team.

**Fig. 1:**
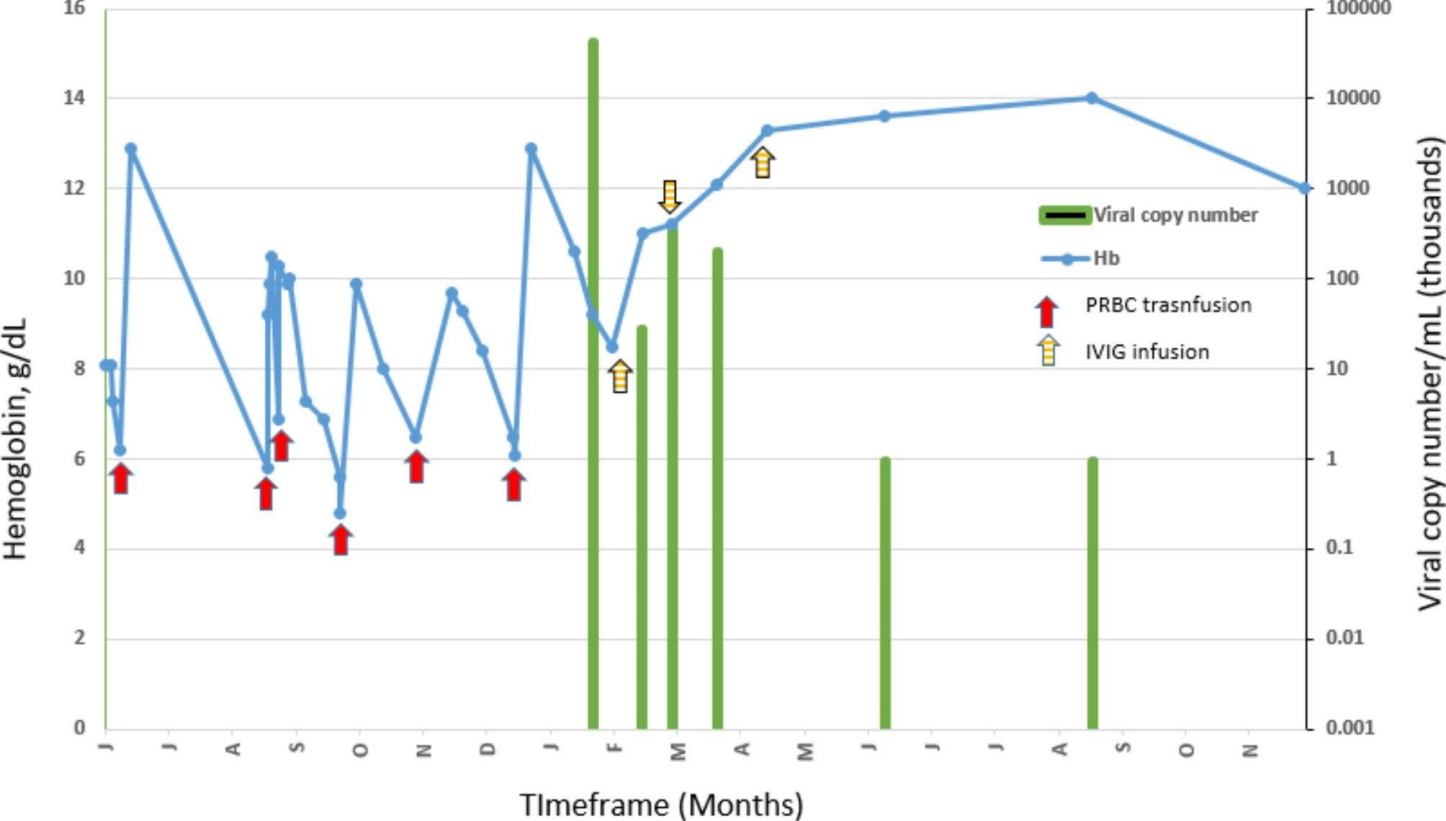
Representation of laboratory test results and interventions

During his outpatient follow-up the child remained anemic with severe reticulocytopenia and required repeated PRBC transfusions. Further investigations did not show any evidence of an inherited bone marrow failure syndrome. His blood PCR for parvovirus B19 was elevated at 4.3 × 10^6^ IU/mL. We opted to treat the child with IVIG and monitor response by parvovirus B19 PCR, blood hemoglobin, and reticulocyte measurements. After three doses of IVIG parvovirus B19 was undetectable in the blood and child became transfusion independent with full recovery of erythropoiesis (See Fig. 1).

## Discussion and conclusions

The most common and serious clinical manifestation of fetal anemia secondary to parvovirus B19 is nonimmune hydrops fetalis. Severe anemia due to immune or non-immune etiologies causing hypoxemia and cardiogenic heart failure may lead to hydrops fetalis and ultimately death in utero [10]. Hydrops fetalis is often diagnosed via prenatal ultrasound as scalp and skin edema, ascites, pleural effusion, placentomegaly, and polyhydramnios [11]. About 90% of hydrops fetalis cases are due to non-immune mediated causes such as parvovirus as preventative measures such as anti-D immune globulin treatment are now in place to decrease immune mediated cases [12].

Parvovirus B19 is highly tropic to human bone marrow and replicates only in erythroid progenitor cells [13, 14]. Resultant pure red cell aplasia is secondary to failure of erythropoiesis. It is a normocytic, normochromic anemia in which the bone marrow is unable to make erythroblasts. In the peripheral blood, there is an absence of reticulocytes. Platelet and leukocyte precursors however, are less likely affected. It has been hypothesized that due to viral induction of apoptosis, parvovirus B19 replication stops the erythrocyte development at the giant pronormoblast stage. Non-erythroid lineages may be affected as well, although these are less efficient in supporting B19 viral replication [13, 15]. Postnatal infections with parvovirus B19 can also cause pure red cell aplasia. Transient erythroblastopenia of childhood is a self-limiting and benign disease which occurs when there is a temporary suppression of erythropoiesis, resulting in reticulocytopenia in the blood [16, 17].

Current management of anemia in children with severe anemia due to parvovirus B19 infection is PRBC transfusion support as needed. Society of Obstetricians and Gynaecologists of Canada guidelines in fact recommend early delivery and intrauterine transfusions as the recommended intervention in hydrops fetalis or fetal anemia following parvovirus infection [18]. Intrauterine transfusions remain the standard of care for non-immune hydrops fetalis associated with severe fetal anemia with significant risk for mortality and morbidity [19–21].

Intravenous immunoglobulins is a group of pooled antibodies from healthy donors with various exposures. Ideally, IVIG contains antibodies against a broad variety of infectious agents, including but not limited to parvovirus. Children, who do not have prior parvovirus exposure and or antibodies against the virus, have no defense mechanism to stop the virus from attacking erythrocytes and causing anemia. IVIG contains neutralizing antibody against parvovirus B19 and has been reported to be effective for chronic B19 infection-related anemia in immunocompromised hosts. It has been used in infants and children with chronic and refractory anemia due to parvovirus infection [5, 22, 23]. In an interesting study, parvovirus antibody enriched immunoglobulins were successfully used in the form of fetal intraperitoneal infusion to treat hydrops fetalis associated with parvovirus B19 infection [24].

IVIG readily crosses the placenta and is available to the fetus. It has also been used successfully for treatment of fetus in the case of hemolytic disease of the newborn due to Rh-alloimmunization. IVIG in this setting is thought to help by diluting maternal antibodies and by inducing competition at the placenta and therefore reducing transplacental transfer of maternal antibodies resulting in lower maternal alloantibody levels and by blocking fetal macrophage function [25, 26]. It would be intriguing to see if IVIG infused to the pregnant woman intravenously would also treat fetal anemia caused by congenital parvovirus infection as IVIG has been successfully used in pregnant woman with minimal adverse effects for a variety of indications [25].

In conclusion, use of IVIG in the setting of congenital parvovirus B19 induced anemia appears to be effective in reducing and potentially eliminating the need for PRBC transfusions. Prenatal IVIG infusion to the mothers of fetuses affected by parvovirus B19 infection to prevent fetal anemia and its potential complications is a subject for future research.

## Data Availability

Data used is not publicly available to protect the personal health information. Fully de-identified clinical test results may be provided upon request.

## Abbreviations

PRBC: packed red blood cell
IVIG: intravenous immunoglobulin
NICU: Neonatal intensive care unit
PCR: Polymerase chain reaction
